# Renal denervation decreases effective refractory period but not inducibility of ventricular fibrillation in a healthy porcine biomodel: a case control study

**DOI:** 10.1186/s12967-014-0367-y

**Published:** 2015-01-16

**Authors:** Jean-Claude Lubanda, Jaroslav Kudlicka, Mikulas Mlcek, Miroslav Chochola, Petr Neuzil, Ales Linhart, Otomar Kittnar

**Affiliations:** 2nd Department of Medicine - Department of Cardiovascular Medicine, First Faculty of Medicine, Charles University in Prague and General University Hospital in Prague, U Nemocnice 2, Prague 2, 128 00 Czech Republic; Institute of Physiology, First Faculty of Medicine, Charles University in Prague, Albertov 5, Prague 2, 128 00 Czech Republic; 3rd Department of Medicine, First Faculty of Medicine, Charles University in Prague and General University Hospital, U Nemocnice 2, Prague 2, 128 00 Czech Republic; Department of Cardiology, Na Homolce Hospital, Roentgenova 2/37, Prague 5, 150 30 Czech Republic

**Keywords:** Renal denervation, Ventricular fibrillation, Electrophysiological study, Biomodel

## Abstract

**Background:**

Ventricular arrhythmias play an important role in cardiovascular mortality especially in patients with impaired cardiac and autonomic function. The aim of this experimental study was to determine, if renal denervation (RDN) could decrease the inducibility of ventricular fibrillation (VF) in a healthy porcine biomodel.

**Methods:**

Controlled electrophysiological study was performed in 6 biomodels 40 days after RDN (RDN group) and in 6 healthy animals (control group). The inducibility of VF was tested by programmed ventricular stimulation from the apex of right ventricle (8 basal stimuli coupled with up to 4 extrastimuli) always three times in each biomodel using peripheral extracorporeal oxygenation for hemodynamic support. Further, basal heart rate (HR), PQ and QT intervals and effective refractory period of ventricles (ERP) were measured. Technical success of RDN was evaluated by histological examination.

**Results:**

According to histological findings, RDN procedure was successfully performed in all biomodels. Comparing the groups, basal HR was lower in RDN group: 79 (IQR 58; 88) vs. 93 (72; 95) beats per minute (p = 0.003); PQ interval was longer in RDN group: 145 (133; 153) vs. 115 (113; 120) ms (p < 0.0001) and QTc intervals were comparable: 402 (382; 422) ms in RDN vs. 386 (356; 437) ms in control group (p = 0.1). ERP was prolonged significantly in RDN group: 159 (150; 169) vs. 140 (133; 150) ms (p = 0.001), but VF inducibility was the same (18/18 vs. 18/18 attempts).

**Conclusions:**

RDN decreased the influence of sympathetic nerve system on the heart conduction system in healthy porcine biomodel. However, the electrophysiological study was not associated with a decrease of VF inducibility after RDN.

## Background

The occurrence of ventricular arrhythmias is influenced by the activity of the autonomic nervous system [1]. The sympathetic nervous system plays an important role in the onset, maintenance and termination of ventricular arrhythmia. Most typically sympathetic activation enhances ventricular arrhythmia while vagal tone suppresses its occurrence. The onset of ventricular arrhythmias is provoked by shortening the effective ventricular refractory time, being a result of sympathetic stimulation. Therefore modulating the activity of the autonomic nervous system might suppress the incidence of ventricular arrhythmias including ventricular fibrillation and provide a rationale for the reduction of the risk of sudden cardiac death. Moreover, RDN was successfully used to reduce the occurrence of electrical storm in many case reports. Impaired cardiac autonomic control and increased sympathetic tone by physical or emotional stress play an important role in the induction of arrhythmia [2] and increase the risk of sudden death [3]. Moreover, increased sympathetic activity as a result of cardiac dysfunction contributes to the worsening of chronic heart failure and increased mortality [4,5]. Therefore the use of beta adrenergic blocking agents is generally recommended as prophylaxis of malignant ventricular arrhythmias to reduce the risk of sudden cardiac death [6,7]. However, some invasive procedures can modulate the tone of sympathetic nerves. Surgical ablation of the lower part of the left ganglion stellate and the first four thoracic ganglia (left cardiac sympathetic denervation) reduces the rate of cardiac events refractory to therapy in most patients with inherited arrhythmia syndromes, such as long-QT syndrome and cathecholaminergic polymorphic ventricular tachycardia (VT) [8]. The potential effects of renal denervation for the reduction of malignant arrhythmia has been described. And its potential benefit is being discussed in heart failure patients with left ventricle dyssynchrony which is independently associated with the risk of sudden cardiac death [9]. Moreover, catheter-based approach has been developed for renal sympathetic denervation (RDN) using several techniques [10]. The ablation procedure reduces sympathetic activation not just in the kidney but also in the whole body [11-13]. Beyond lowering of blood pressure RDN was shown to reduce the resting heart rate and to prolong PR interval in patients with resistant arterial hypertension [14]. Also in a pig model of atrial fibrillation RDN reduced duration of pacing-induced episodes of the arrhythmia [15]. Moreover, stimulation of left stellate ganglion and rapid atrial pacing in animals after RDN did not increase the inducibility of atrial fibrillation [16]. Similar findings were described in patients after pulmonary vein isolation along with RDN who experienced significantly fewer episodes of atrial fibrillation at follow-up than patients after pulmonary vein isolation alone [17]. Focused on ventricular arrhythmias, Linz et al. [18] reported a significant reduction of spontaneous ventricular extrabeats and ventricular fibrillation (VF) episodes during acute myocardial ischemia and reperfusion in dogs after RDN. According to case reports, a significant decrease of VT/VF was observed in two patients with dilated and hypertrophic cardiomyopathy and in a patient after acute myocardial infarction who all suffered from treatment-resistant electrical storms [19,20]. The objective of this experimental study was to compare changes of electrophysiological parameters and inducibility of VF in a healthy porcine biomodel 40 days after RDN with a control group.

## Methods

**Figure 1:**
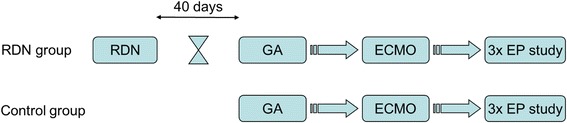
**Overall scheme of the study.** RDN indicates renal denervation; GA, general anesthesia; ECMO, extracorporeal membrane oxygenation; EP, electrophysiological.

The experimental protocol was designed to perform the controlled study in 6 biomodels 40 days after RDN (RDN group) and in 6 healthy animals (control group). The electrophysiological (EP) study was performed using catheter based techniques. The peripheral veno-arterial extracorporeal membrane oxygenation (ECMO) was used because of myocardial protection during the ventricular pacing and arrhythmias. An overall scheme of the study is shown in Figure 1.

Cross-bred swine (Landrace x White; 44 ± 3 kg) were used for the experiments in an accredited experimental university laboratory by a skilled team of clinicians and veterinary specialists. The animals were handled in accordance with the guidelines of research animal use [21]. The protocol was approved by the Charles University 1st Medical School Institutional Animal Care and Use Committee and performed at the Animal Laboratory, Institute of Physiology, 1st Medical School, Charles University in Prague in accordance with Act No 246/1992 as amended, Collection of Laws, Czech Republic, that is harmonized with EU Directives 86/609/EEC as amended, 2007/526/ES, 2010/63/EU.

### Anesthesia and monitoring

The pigs were sedated by intramuscular application of azaperone (2–3 mg/kg) and ketamine (20 mg/kg). Then the marginal ear vein was cannulated and general anesthesia was induced by intravenous bolus of propofol (1–2 mg/kg). After preoxygenation via facial mask, orotracheal intubation was performed. During the experiment were the biomodels mechanically ventilated using Intellivent-ASV closed-loop system (G5, Hamilton Medical, Bondauz, Switzerland) to maintain normoxia (SpO_2_ 98%) and normocapnia (EtCO_2_ 38–40 mmHg) respecting the actual metabolic rate. The total intravenous anesthesia was maintained by continuous administration of propofol (6–12 mg/kg/h) and morphine (0.1-0.2 mg/kg/h). The depth of anesthesia was regularly assessed by the photoreaction and the corneal reflex and adjusted accordingly. Intravenous infusion of Ringer’s solution was given to reach and maintain central venous pressure between 6 and 8 mmHg. Anticoagulation was provided by unfractionated heparin bolus (100 IU/kg IV) followed by continuous intravenous drip (40–50 IU/kg/h) to maintain target activated clotting time 180–250 seconds (values checked every hour with Hemochron Junior+, International Technidyne Corporation, Edison, NJ, USA). Sheaths and catheters were inserted to femoral and carotid/jugular vessels as needed. Invasive blood pressure from carotid and pulmonary artery, central venous pressure (TruWave, Edwards Lifesciences, USA), body surface ECG, capnometry and pulse oximetry were continuously monitored by bedside monitor (Life Scope TR, Nihon Kohden, Japan).

### Renal denervation

After initiation of general anesthesia and mechanical ventilation an 8 french sheath was placed into the right common femoral artery under ultrasound guidance and consequently another 6 french sheath was introduced into the right common femoral vein. The later was used for the acquisition of samples for biochemical analysis during the procedure. According to the protocol, after preliminary samples were withdrawn, renal angiography was performed and an ablation catheter was then introduced into the renal arteries. In 4 cases, the EnligHTN catheter (St. Jude Medical, USA) was used and in 2 cases we used the Symplicity catheter (Medtronic, USA). The ablation procedure was performed according to the instruction for use of each device in both arteries in each case and the effect of the procedure was controlled by a drop of impedance of at least 10%. At the end of RDN procedure, the femoral sheath was extracted and the groin was surgically sutured. Subsequently, the administration of anesthetics and analgesics was stopped and successful weaning from artificial ventilation was achieved. Then were the animals extubated. For next 40 days, the animals were bred in a certified menagerie.

### Cardiopulmonary bypass

Before starting the EP study, cardiopulmonary bypass (CPB) was established: an inlet 19 F cannula was inserted via femoral vein into the right atrium and an outlet 17 F cannula into abdominal aorta via femoral artery. The cannulae were connected to ECMO circuit consisting of a blood pump (Levitronix Centrimag, Levitronix, USA) and oxygenator (Quadrox, Maquet, Germany). The ECMO circuit was primed with 500 mL of saline with 2500 IU of unfractionated heparin. The ECMO blood flow was empirically set at 40 mL/kg/min to prevent low cardiac output and hypotension during the ventricular stimulation. After the onset of VF, the flow was increased to 80-100 mL/kg/min as needed to keep mean arterial pressure of 60 mmHg until an effective pulsatile sinus rhythm with mean arterial pressure above 60 mmHg was restored. Ventilation support, as well as ECMO gas flow, was regularly adjusted to reach the target values (SpO_2_ 98% and pCO_2_ 38-40 mmHg).

### Electrophysiological study

**Figure 2:**
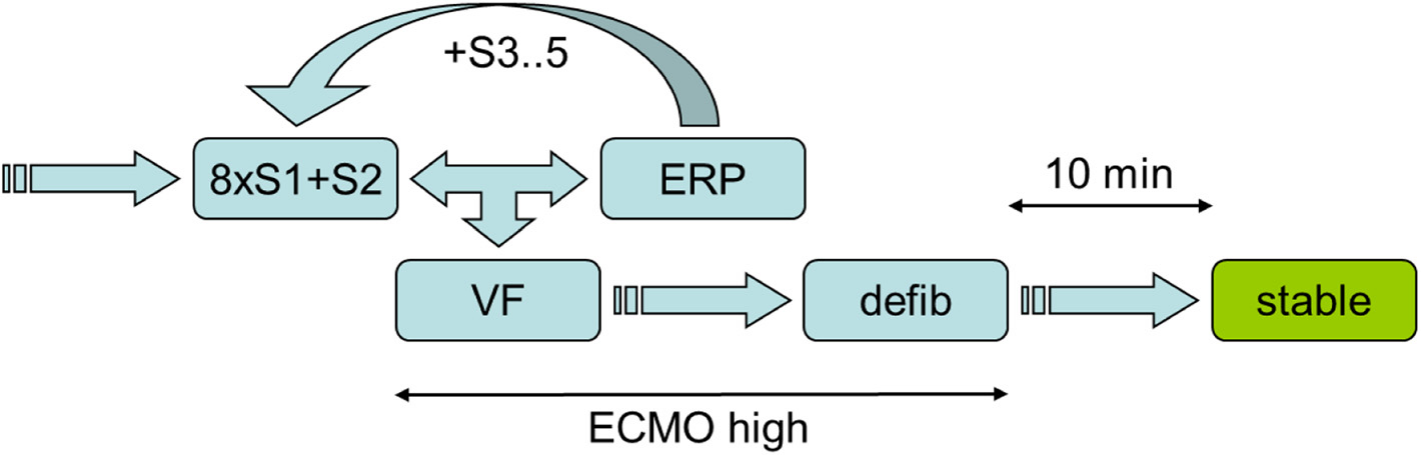
**Scheme of electrophysiological study.** 8xS1 indicates the train of eight basic stimuli; S2-5, up to five extrastimuli; ERP, effective refractory period; VF, ventricular fibrillation; defib, defibrillation; ECMO high, increasing of the ECMO flow to 80-100 mL/kg/min after onset of VF until the sinus rhythm was restored.

A diagnostic decapolar catheter (Response CSL, St. Jude Medical, USA) was inserted under fluoroscopic guidance into the apex of the right ventricle (RV) via right jugular vein to provide monitoring of intracardial electrograms and to induce VF. Five bipolar channels were recorded from the apex to base of RV at 3 kHz sampling rate. VF was induced by programmed ventricular stimulation (PVS, Figure 2). Briefly, electrical stimuli (12 ms duration, maximal stimulation current 20 mA) were delivered from the apex of RV. Eight basic stimuli (S1) of cycle length 300, 350 or 400 ms according to the heart rate were coupled with up to 4 extrastimuli (S2-S5). The coupling interval of S2 was decreased at 10 ms steps until the ventricular effective refractory period (ERP) was achieved. S2 coupling interval was then set to 10 ms above ERP and analogically S3 or S4 stimuli were delivered until VF was reached. There were at least 10 s intervals between pacing sequences. VF was defined as an orderless rhythm without detectable QRS complexes lasting longer than 30 s. Normal SR was restituted by the transcutaneous defibrillation using pads placed in the conventional position sternum - apex (TEC-5521, Nihon Kohden, Japan). Biphasic shocks with rising energy (150-200-270 J) were delivered as needed. Another PVS procedure was performed at least 10 minutes after SR restoration in a previous one.

### Data acquisition protocol

The baseline surface ECG was acquired during 10 minutes at minimum of 60 minutes after general anesthesia initiation. Heart rate (HR), PQ and QT intervals were assessed ten times (in each minute of the ECG record) by offline manual analysis with appropriate time resolution and QTc was calculated using Bazett’s formula (QT interval divided by square root of RR interval). Then was CPB established and ECMO connected. EP study began at minimum of 30 minutes after connecting ECMO. PVS procedures were performed three times in each animal. Finally, an autopsy was carried out to obtain samples of renal arteries and macroscopic and histological assessment of the arterial wall and perirenal nerves. All procedures were provided by a skilled pathologist.

### Statistics

The sample size was calculated using Medcalc software (Medcalc® Version 12,1.4.0) based on the primary outcome of the study which was the inducibility of VF. The number of attempts to induce VF was estimated according the study by linz et al. [18] where VF occurred in 14% of denervated pigs and in 83% of sham treated pigs. For each experiment, we planned 3 attempts. The minimal required sample size per group for equal samples sizes for alpha = 0.05 and power = 0.80 (beta = 0.20) was 5, meaning 15 attempts per group assuming that the data will be approximately normally distributed. Data are presented as medians (interquartile ranges). The nonparametric unpaired Mann–Whitney test was used to assess the differences. The *P* values < 0.05 were considered significant. Statistical analysis and graphs were performed using Prism 5.0 (GraphPad, La Jolla, CA, USA).

## Results

**Figure 3:**
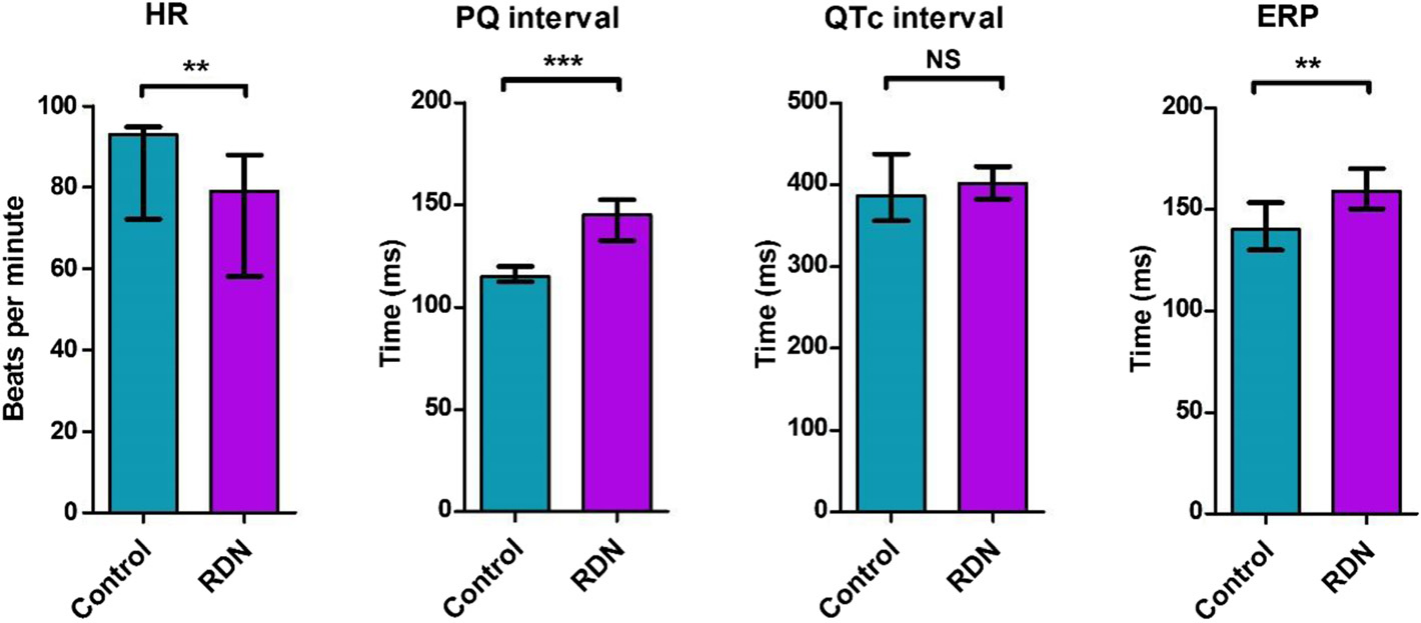
**Comparison of electrophysiological parameters of RDN and control group.** HR indicates heart rate; ERP, effective refractory period of ventricles; VF, ventricular fibrillation; Control, control group; RDN, RDN group; NS, statistically non-significant; ** P < 0.01; P *** P < 0.0001.

**Figure 4:**
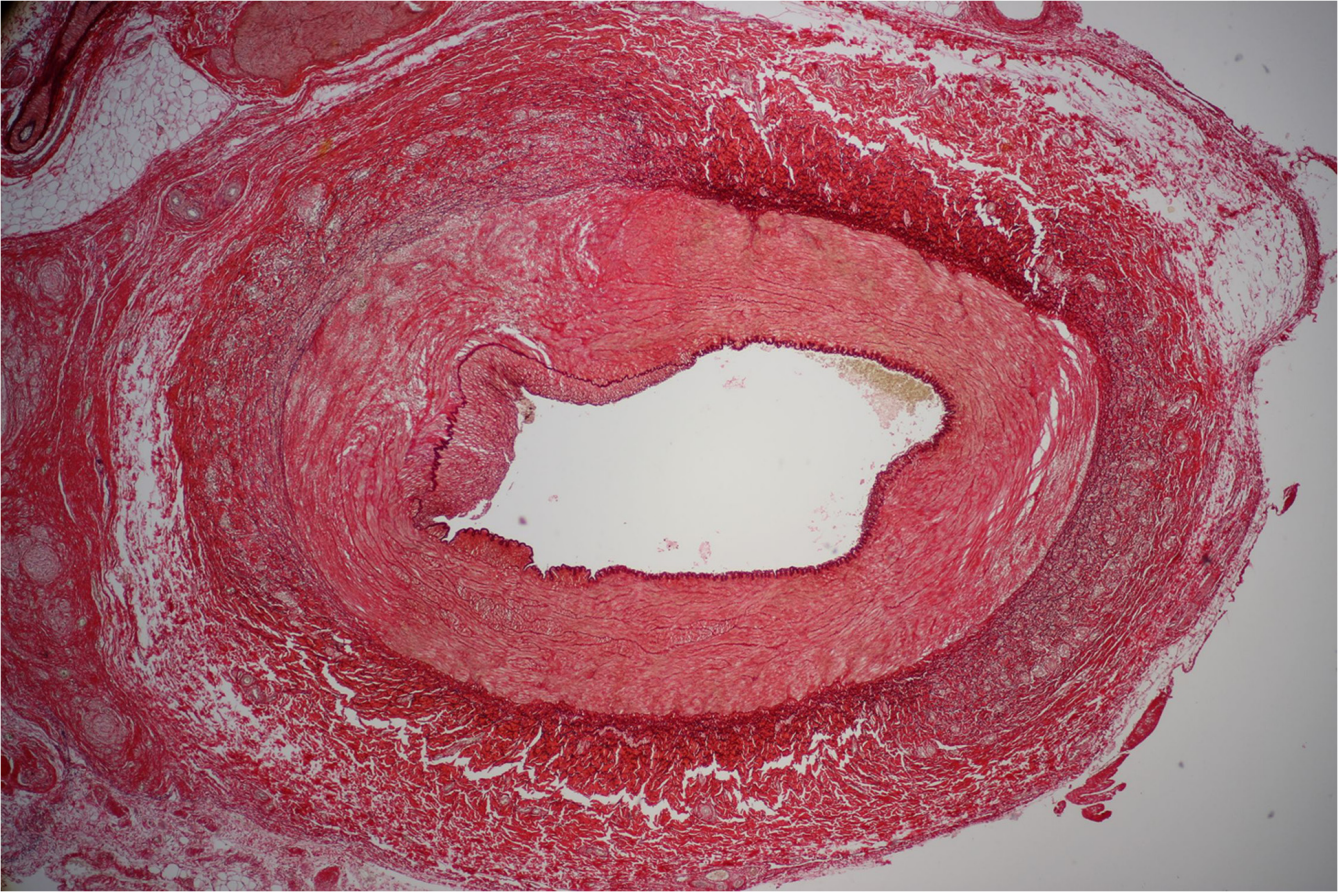
**Histological finding of renal artery 40 days after successful renal denervation.** In the left portion of the image extensive fibrosis of the intima, the media and fragmentation of the lamina elastica externa and the adventicia depicted.

The electrophysiological parameters and VF inducibility data are summarized in Table 1 and Figure 3. According to histological findings, destruction of perirenal nerves was successful in both arteries in each case regardless of used ablation catheter (Figure 4). No arterial stenosis or endothelial damage was found 40 days after RDN.

**Table 1:** **Comparison of ECG and ventricular fibrillation (VF) induction parameters of RDN and control group**

**Control group**						
**Animal #**	**HR (ms)**	**PQ (ms)**	**QTc (ms)**	**Induction parameter S1-S5 (ms)**	**ERP (ms)**	**Inducibility of VF**
1	89 (88; 91)	114 (113; 117)	361 (357; 364)	400/200/140/110/100	166	3/3
2	94 (93; 94)	118 (116; 118)	359 (356; 360)	350/150/110	140	3/3
3	96 (96; 98)	120 (116; 123)	438 (436; 444)	350/160/120/90	163	3/3
4	72 (72; 73)	113 (113; 113)	368 (367; 368)	350/140/60	130	3/3
5	95 (95; 95)	110 (108; 110)	353 (351; 355)	350/150/140	140	3/3
6	68 (67; 68)	143 (140; 144)	328 (326; 332)	300/120/60	128	3/3
**Total**	**93 (72; 95)**	**115 (113; 120)**	**386 (356; 437)**		**140 (133; 150)**	**18/18 (100%)**
**RDN group**						
**Animal** #	**HR (ms)**	**PQ (ms)**	**QTc (ms)**	**Induction parameter S1-S5 (ms)**	**ERP (ms)**	**Inducibility of VF**
1	78 (78; 79)	123 (120; 124)	386 (383; 391)	400/190/170	166	3/3
2	81 (78; 85)	146 (143; 148)	389 (382; 400)	400/170/140	149	3/3
3	88 (87; 89)	149 (145; 153)	472 (466; 475)	350/200/80	190	3/3
4	49 (48; 49)	161 (158; 163)	356 (354; 358)	400/200/160	166	3/3
5	113 (113; 113)	131 (128; 133)	421 (416; 425)	350/180/100	170	3/3
6	58 (58; 59)	153 (150; 155)	405 (402; 407)	350/160/140	150	3/3
**Total**	**79 (58; 88)** ^**^	**145 (133; 153)** ^***^	**402 (382; 422)** ^**NS**^		**159 (150; 169)** ^**^	**18/18 (100%)**

Induction parameters represent minimal cycle lengths of basic stimuli and up to five extrastimuli (S1-5). In each animal was EP protocol made three times. HR indicates heart rate; ERP, effective refractory period of ventricles; VF, ventricular fibrillation; ^NS^,statistically non-significant; **P < 0.01; P ***P < 0.0001.

### ECG changes at baseline

Comparing RDN and control group, basal HR was significantly lower in RDN group: 79 (58; 88) vs. 93 (72; 95) beats per minute, p = 0.003. Accordingly, PQ interval in RDN group was longer: 145 (133; 153) vs. 115 (113; 120) ms, p < 0.0001, and QTc intervals were comparable between RDN and control group: 402 (382; 422) vs. 386 (356; 437) ms, p = 0.1.

### Inducibility of VF

VF was induced using the same protocol in each attempt in all animals of both RDN and Control groups (18/18 vs. 18/18) and also the median number of extrastimuli did not differ. However, ERP was significantly prolonged in RDN group: 159 (150; 169) vs. 140 (133; 150) ms, p = 0.001.

## Discussion

In this experimental study we have shown that RDN changed autonomic control of the heart at interval of 40 days. Decreased sympathetic activity was observed as pleiotropic changes in the heart conduction system, e.g. decrease of the rest heart rate, prolongation of atrioventricular conduction and increase of ERP of ventricles.

The prolongation of ERP is a sign of lower ventricular myocardial excitability. By analogy with former experimental studies in dogs, surgical excision of the left stellate ganglion prolonged ERP of 4–7 ms [22] and raised the VF threshold [23]. This indicates that despite ablation of perirenal sympathetic nerves, RDN has a similar effect as the direct destruction of heart-related sympathetic ganglia and nerves. Moreover, the antiarrhythmic effect of RDN seems to be comparable to stimulation of the local parasympathetic system. As described by André et al. [24], stimulation of the vagal nerve on isolated rabbit hearts in vitro prolonged ERP by 13% (vs. 14% in this study) and VF threshold was significantly higher. In vivo data demonstrated the anti-arrhythmic effect of vagal stimulation especially in preventing decrease of VF threshold during the stimulation of the sympathetic nerves [25].

Nevertheless, using the same PVS protocol we did not prove the benefit of RDN in terms of decrease in VF inducibility in this experimental setting (normal porcine heart). According to previously published clinical observational data by Bourke et al. [26], thoracic epidural anesthesia or surgical left cardiac sympathetic denervation allowed to reduce the incidence of ventricular tachycardia (VT) by 68% in 14 patients with structural heart disease refractory to pharmacotherapy and catheter ablation. As mentioned above, Linz et al. [18] reported a significant reduction of spontaneous ventricular extrabeats and VF episodes during acute myocardial ischemia and reperfusion in dogs after RDN. On the other hand, they also discussed that using a beta-blocking agent (atenolol) showed a comparable effect. In case reports by Ukena [19] and Hoffmann [20] concerning patients with dilated/hypertrophic cardiomyopathy or after acute myocardial infarction, RDN was effective in decreasing VT/VF events in addition to excessive antiarrhythmic medical and ablation therapy.

We can hypothesize that the influence of RDN could be more pronounced during the acute stress reaction or in the presence of an arrhythmogenic substrate (e.g. post-infarction scar) than in normal state. Also, our study cannot address the superiority of RDN compared to the use beta-blockers and other antiarrhythmic drugs.

As mentioned above, the study was limited by using healthy animals not considering any predisposition to ventricular arrhythmias like ischemic-reperfusion injury or structural changes of the myocardium. Further, electrophysiological parameters could be influenced by depth of analgosedation, despite the effort to maintain the same depth of anesthesia and similar dosage of propofol and morphine. Also the number of tested experimental animals was low and the electrophysiological study was not performed in the same biomodel before and after RDN because of presumed high morbidity-mortality of biomodels during the 40-day period after the procedure. Thus, the translation of the results to humans should be interpreted with caution despite the fact, that our biomodels were represented by a breed repeatedly validated for simulation of human cardiac arrest and evaluation of VF [27,28].

## Conclusions

We can conclude that renal denervation affected the autonomic heart control significantly in favor of decreased tone of the sympathetic nervous system in a healthy porcine biomodel 40 days after RDN procedure. Unfortunately, these changes were not associated with lower ventricular fibrillation inducibility using the same PVS protocol in comparison with the control group.

## Abbreviations

CPB: Cardiopulmonary bypass
ECMO: Extracorporal membrane oxygenation
EP: Electrophysiological study
ERP: Effective refractory period
HR: Heart rate
PVS: Programmed ventricular stimulation
RDN: Renal denervation
RV: Right ventricle
VF: Ventricular fibrillation
VT: Ventricular tachycardia
